# *KRAS* Mutation Detection in Paired Frozen and Formalin-Fixed Paraffin-Embedded (FFPE) Colorectal Cancer Tissues

**DOI:** 10.3390/ijms12053191

**Published:** 2011-05-17

**Authors:** Jérome Solassol, Jeanne Ramos, Evelyne Crapez, Majda Saifi, Alain Mangé, Evelyne Vianès, Pierre-Jean Lamy, Valérie Costes, Thierry Maudelonde

**Affiliations:** 1 Department of Cellular Biology, Center Hospital University, Montpellier 34000, France; E-Mails: a-mange@chu-montpellier.fr (A.M.); e-vianes@chu-montpellier.fr (E.V.); t-maudelonde@chu-montpellier.fr (T.M.); 2 University of Montpellier I, Montpellier 34000, France; E-Mails: j-ramos@chu-montpellier.fr (J.R.); v-costes_martineau@chu-montpellier.fr (V.C.); 3 Department of Pathology, Center Hospital University, Montpellier 34000, France; E-Mail: m-saifi@hotmail.fr; 4 Department of Biology, Centre Lutte Contre Cancer Val d’Aurelle, Montpellier 34000, France; E-Mails: ecrapez@valdorel.fnlcc.fr (E.C.); Pierre-Jean.Lamy@valdorel.fnlcc.fr (P.-J.L.)

**Keywords:** genotyping, *KRAS*, fixative

## Abstract

*KRAS* mutation has been unambiguously identified as a marker of resistance to cetuximab-based treatment in metastatic colorectal cancer (mCRC) patients. However, most studies of *KRAS* mutation analysis have been performed using homogenously archived CRC specimens, and studies that compare freshly frozen specimens and formalin-fixed paraffin-embedded (FFPE) specimens of CRC are lacking. The aim of the present study was to evaluate the impact of tissue preservation on the determination of *KRAS* mutational status. A series of 131 mCRC fresh-frozen tissues were first analyzed using both high-resolution melting (HRM) and direct sequencing. *KRAS* mutations were found in 47/131 (35.8%) using both approaches. Out of the 47 samples that were positive for *KRAS* mutations, 33 had available matched FFPE specimens. Using HRM, 2/33 (6%) demonstrated suboptimal template amplification, and 2/33 (6%) expressed an erroneous wild-type *KRAS* profile. Using direct sequencing, 6/33 (18.1%) displayed a wild-type *KRAS* status, and 3/33 (9.1%) showed discordant mutations. Finally, the detection of *KRAS* mutations was lower among the FFPE samples compared with the freshly frozen samples, demonstrating that tissue processing clearly impacts the accuracy of *KRAS* genotyping.

## 1. Introduction

Over the past decade, with the introduction of new cancer drugs such as targeted agents, the treatment of metastatic colorectal cancer (mCRC) has greatly improved. The epidermal growth factor receptor (EGFR) is a key molecular player in cell growth and survival. This receptor is often overexpressed in mCRC and contributes to cancer progression through the modulation of biological events, such as proliferation, adhesion and angiogenesis. Monoclonal antibodies such as cetuximab prevent ligand-induced EGFR activation and the subsequent induction of signal transduction pathways, thus disrupting downstream signaling, adhesion, and angiogenetic pathways. Cetuximab has been shown to be clinically effective in phase II trials for treating irinotecan-refractory mCRC patients who present positive EGFR expression [1,2]. Recent randomized phase III clinical trials have also shown that cetuximab has significant clinical activity when administered in combination with irinotecan as a first- or second-line agent [3]. A significant proportion of EGFR-positive mCRC patients, however, are resistant to anti-EGFR treatments. No more than 23% of mCRC patients respond to the combination treatment of cetuximab and irinotecan, and less than 10% respond to anti-EGFR monotherapy [4,5]. One reason for this difference is that other pathways may also be activated downstream of EGFR because of a mutation in the *KRAS* oncogene [6,7]. *KRAS* gene mutations at codons 12 (wild-type GGT) and 13 (wild-type GGC) have been shown to be predictive of the response to cetuximab in mCRC [8] and to behave as independent prognostic factors in advanced mCRC with cetuximab treatment [6].

For ethical and economic reasons, it is necessary to better define the subpopulation of patients who would truly benefit from cetuximab through *KRAS* mutation analysis. Beyond the available molecular methodology (*i.e*., High Resolution Melting (HRM) or direct sequencing), the optimal consideration for routine identifying *KRAS* mutations is in the tissue source. Fresh-frozen tissue represents an ideal supply of archival material for molecular investigations but is not usually possible in routine practice. Formalin-fixed paraffin-embedded (FFPE) tissues undergo effective preservation of the cellular, architectural, and morphological details and allow easy storage at room temperature for extensive periods. For these reasons, this processing has become the principal method for archiving tissues to determine *KRAS* status. However, FFPE processing impairs the extraction efficacy and quality of DNA, thus preventing the ability to conduct high-quality molecular analyses and potentially affecting the results of the *KRAS* analysis [9–17]. The main objective of this study was to examine whether *KRAS* genotyping on FFPE CRC specimens give comparable results with freshly frozen specimens simultaneously obtained from the same patient. To meet this objective, we compared the *KRAS* status between the paired freshly frozen and FFPE tissue samples using both a screening and a diagnostic PCR-based method.

## 2. Results and Discussion

**Figure 1.**
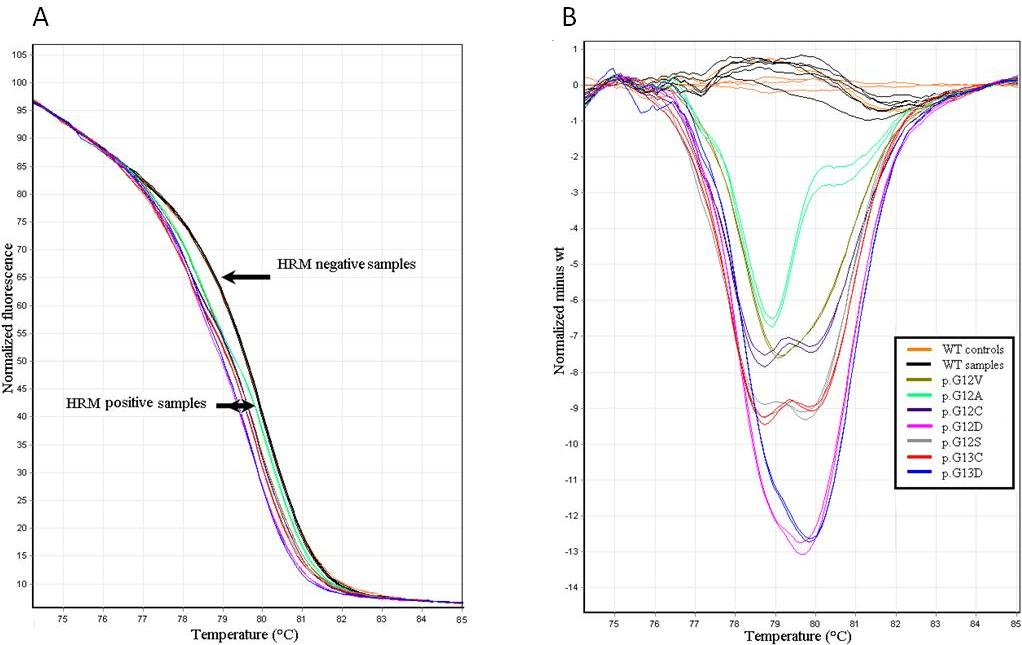
High-resolution melting (HRM) analysis of exon 2 of *KRAS* in 10 DNA specimens from frozen samples. (**A**) Normalized high-resolution melting curves. PCR products were labeled with an intercalating dye, and the fluorescence signal was plotted as the temperature increased; (**B**) The difference plot displays the melting curve of each tested sample subtracted from the reference curve obtained by analyzing a control wild-type *KRAS* sequence.

First, we retrospectively analyzed mutations in exon 2 of *KRAS* in a series of 131 frozen mCRC tumor samples using HRM analysis. The genomic yield of DNA obtained from the frozen tissue samples was 798.9 ± 826.9 μg/mL. PCR inhibition was not observed for any of the samples, and therefore, PCR was completed for all of the tested DNA samples. Starting with 25 ng of genomic DNA as a template, the mean threshold cycle value (Ct) was 21.79 ± 1.62 (range: 19.68–28.85). The melting curve obtained for the 84-bp amplicon was monophasic (Figure 1A), which suggested only one homogeneous melting domain and allowed a reliable distinction of mutated samples. In particular, for 47 (35.8%) specimens of the series, a distinct shape of the curves on normalized difference plots was observed, and the corresponding curve patterns for the HRM difference plots unambiguously revealed the HRM-positive samples. The difference plots for exon 2 of *KRAS* in 7 HRM mutation-positive (related to p.G12A, p.G12C, p.G12S, p.G12D, p.G12V, p.G13C and p.G13D) and 3 HRM mutation-negative samples are shown in Figure 1B.

Exon 2 of *KRAS* was analyzed in the same 131 samples by direct sequencing. Long (245 bp) DNA fragments were successfully amplified from all of the frozen samples. The HRM-determined status of exon 2 of *KRAS* was confirmed by direct sequencing for all of the samples. Eleven different *KRAS* mutations were observed among the 47 HRM-positive samples, with p.G12D, p.G12V and p.G13D representing the most frequent substitutions at frequencies of 31.9%, 27.7%, and 17%, respectively (Table 1). In addition, one sample exhibited a double point mutation that combined the p.G12V alteration with a silent mutation in codon 13. As expected, all of the HRM-negative samples carried the wild-type sequence of exon 2 of *KRAS*.

**Figure 2.**
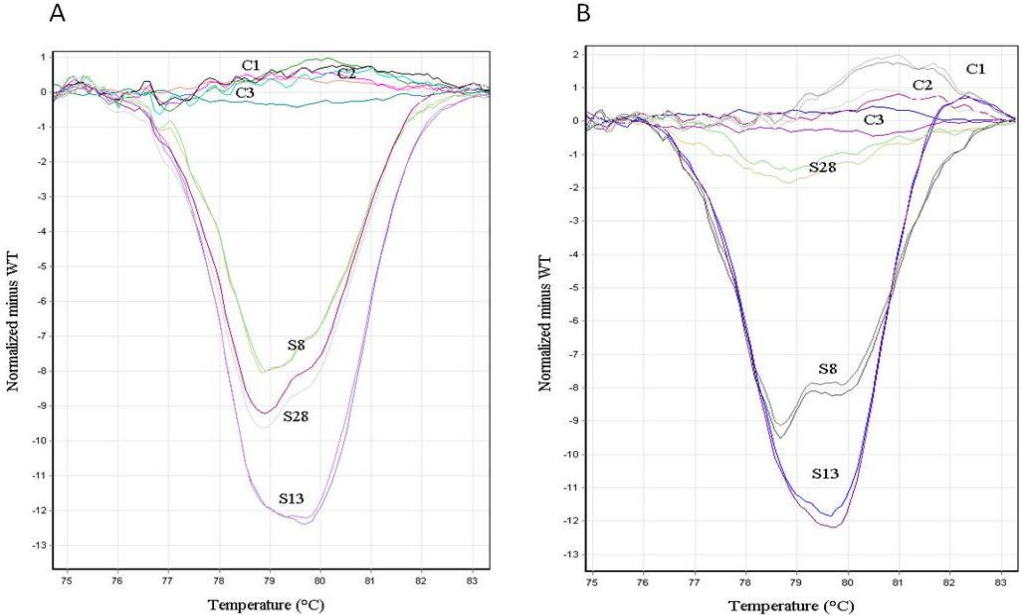
The HRM profiles of frozen tissue samples and their matched FFPE samples. (**A**) The HRM profiles of three mutated *KRAS* frozen samples (S8, S28, and S13) and three wild-type *KRAS* frozen samples (C1, C2, and C3) are shown; (**B**) The HRM profiles of S8, S28, S13, C1, C2, and C3 matched FFPE samples are shown.

Among the 84 frozen DNA samples considered to have wild-type *KRAS* by direct sequencing and HRM, 68 matched FFPE samples with more than 30% tumor cells were available. Using HRM, all of these samples showed the wild-type *KRAS* genotype. Except for four samples that were not amplified, all of these samples showed the wild-type *KRAS* genotype using direct sequencing. Among the 47 frozen DNA samples considered to have *KRAS* mutation by direct sequencing and HRM, only 33 matched FFPE samples with more than 30% of tumor content were available. A high yield of DNA (722.6 ± 406 μg/mL) was obtained, and no substantial differences in the yield of DNA were observed compared with that of the frozen tissue samples. For the HRM analysis, a shift toward higher quantification cycle (Cq) values (mean: 29.54 ± 1.3) and a larger Cq range (25.4–31.95) were observed for the FFPE specimens compared with the frozen specimens. Two of 33 (6%) tested genomic DNA samples demonstrated an ineffective amplification (Cq > 33). Both the normalized curves and difference plots showed a profile similar to that observed with wild-type *KRAS* for two others (6%) specimens (Figure 2), resulting in a total of 4/33 (12.2%) discordant results between the paired frozen and FFPE samples (Table 2).

**Figure 3.**
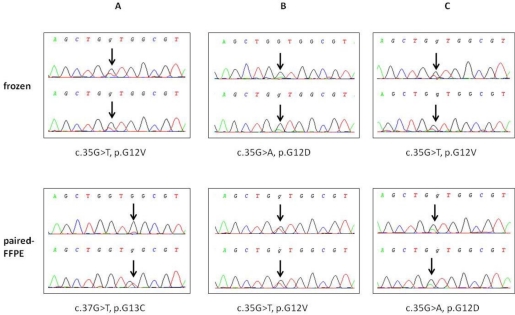
Discordant electropherograms of exon 2 of *KRAS* obtained by direct sequencing between 3 paired frozen and FFPE specimens. The upper panel shows the electropherograms obtained for frozen samples 8 (**A**), 29 (**B**), and 127 (**C**) using the forward (upper) and reverse (bottom) primers. The bottom panel shows the discordant nucleotide alterations observed in the matched FFPE specimens.

In the direct sequencing, the 245-bp PCR product was successfully amplified in only 27/33 (81.8%) cases. Given that the rate of successful PCR amplifications is known to be at least partially related to the size of the product [14,18–20], we designed a new set of primers to reanalyze exon 2 of *KRAS*. By reducing the size of the amplified DNA fragment from 245 bp to 164 bp, the success rate was increased to 31/33 (94%) (Table 2). Among them, 6/33 (18.1%) exhibited a wild-type *KRAS* status, and 3/6 were also found to have a wild-type status or were not amplified with HRM, demonstrating that direct sequencing is a less sensitive method for mutation detection (Table 2). Notably, when we compared the type of nucleotide changes in the mutated *KRAS* between the paired frozen samples and FFPE specimens, discordant nucleic alterations were discovered in 3/33 (9.1%) samples (G12V, G12D, and G12V in frozen and G13C, G12V, and G12D in paired FFPE samples, n°8, 29, and 127, respectively) (Figure 3). Finally, in one case (n°131) that had a double mutation 35_36GT > TC, direct sequencing was able to detect the nucleotide change, unlike HRM, which exhibited a wild-type profile for that case.

The potential benefit of *KRAS* status determination in mCRC is clear. Patients without *KRAS* mutations in codons 12 and 13 exhibit a significant antitumor response in a treatment regimen that includes cetuximab compared with patients who are not treated with cetuximab [21]. Therefore, the U.S. Food and Drug Administration and the European Medicines Agency have mandated that the mutational status of *KRAS* be determined prior to anti-EGFR treatment. However, no standard recommendations have been proposed for the management of CRC specimens that will allow the determination of *KRAS* status.

The impact of specimen processes and storage on the accuracy of HRM and direct sequencing of *KRAS*, to our knowledge, has never been systematically investigated. Some previous studies determined *KRAS* and *EGFR* genotypes in both paraffin-embedded and frozen tissues [14,18,22–26]. However, most studies used specimens from different patients and do not compare the same tissue sample divided into two parts [22–26]. Thus, the same tumor materials may not be available, making genotype comparisons difficult. In our study, 131 tissue samples were processed under freshly frozen and FFPE conditions in parallel. With an average *KRAS* mutation frequency of 35.8% in the frozen tissues, our results were consistent with previously published reports [8,21,27,28]. In these mutation-positive specimens, the genotype determined using HRM and direct sequencing was fully concordant, demonstrating that HRM remained a confident screening strategy for *KRAS* mutation detection, as recently reported in several studies [10,13,17,22,29–33]. Importantly, although attention was paid to avoid false-negative results caused by amplification of normal cells by including only specimens with more than 30% tumor cells, 6% of the FFPE samples were considered mutation-positive in the matched frozen samples that were identified as wild-type by HRM. This value was higher using direct sequencing. The relatively high degree of false-negative detection may be explained by the low sensitivity of both methods, particularly direct sequencing, to detect DNA variation. No correlation was observed between the FFPE and matched frozen samples with regard to the percentage of tumor cells, which was estimated to be between 30% and 90% in the analyzed samples. However, this absence of correlation can certainly also be attributed to the direct impact of tissue processing on the accuracy of *KRAS* genotyping. Finally, Figure 3 showed discrepant *KRAS* nucleotide changes in three samples due to the conservation process. However, these changes did not modify the *KRAS* genotype interpretation. Indeed, the tumor was still mutation-positive, and the patient in both cases was ineligible for treatment with anti-EGFR antibodies.

**Figure 4.**
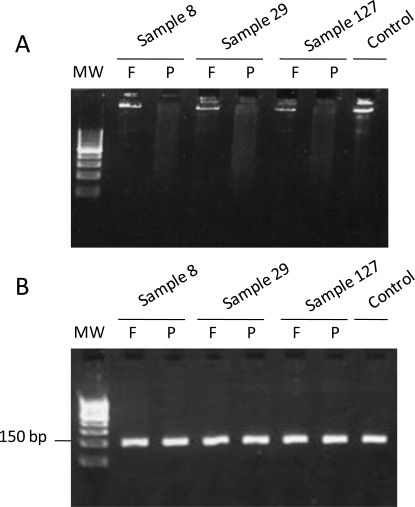
DNA (**A**) and KRAS 164-bp PCR products (**B**) run on 2% agarose gel for samples 8, 29, and 127 frozen of fixed in formaldehyde. Non-degraded DNA exhibited bands of high molecular weight. DNA extracted from blood samples were used as a positive control. MW: molecular weight. F: frozen samples. P: FFPE samples.

Evaluation of the degree of DNA degradation (preservation) is of major importance when handling FFPE samples; otherwise, real-time PCR and sequencing results may not be interpreted appropriately. In our study, we performed a 2% agarose gel electrophoresis to check the DNA degradation level in each sample. As expected, the frozen samples were not degraded, whereas the FFPE samples were partially fragmented. However, when we checked for PCR product amplifications, we observed a correct amplification in both the FFPE and frozen tissues, demonstrating that our PCR conditions were adapted to the FFPE samples. We showed examples of DNA fragmentation (Figure 4A) and *KRAS* 164-bp PCR products (Figure 4B) in 3 FFPE and matched frozen samples with discrepant nucleotide changes.

Several studies have investigated the sample quality requirements of FFPE tissues for sequencing approaches [14,16,34,35]. Interestingly, Miyamae and collaborators adapted the Smart Amplification Process version 2 to rapidly detect *EGFR* and *KRAS* mutations in DNA extracted from FFPE tissues [14]. That study clearly demonstrated that this procedure could identify mutations with high accuracy and gave a reliable diagnostic result based exclusively on amplification [14]. In addition, Troncone and collaborators also proposed options for testing the degree of FFPE DNA preservation and amplification capacity, such as the inclusion of internal controls within qPCR reactions. In cases where specimens are not available, *KRAS* testing may be reliably performed on cytological specimens [16].

In our series, we observed that direct sequencing revealed novel nucleotide changes in three FFPE samples compared with their respective frozen samples (Table 2). Using the same molecular assay, Marchetti *et al*. found 45 artifactual mutations in exons 18 through 21 of the *EGFR* gene from 10 independent PCR amplification products of 70 lung cancer FFPE sections [36]. The authors demonstrated that artifactual C > T/G > A or A > G/T > C transitions, which we also observed in our study, appeared in the DNA isolated from paraffin-embedded tissue samples. These artifacts were ascribed to postmortem deamination of cytosine or adenine to uracil or hypoxanthine residues, respectively. Recently, Gallegos Ruiz *et al*. compared *EGFR* mutations in 47 non-small cell lung cancer samples in frozen and paraffin-embedded specimens [18] and detected significant nucleotide changes in FFPE samples, not in frozen specimens. Overall, these results provide evidence for the influence of fixation and embedding procedures on the appearance of artifactual mutations or false-negative results. It is likely that deamination does not occur uniformly throughout the tumor, but at different sites. However, we did not check the possible intra-tumor heterogeneity of this DNA modification by performing DNA sequencing at multiples sites. This issue should be considered a limitation of our study. Formalin has been used for decades as the most abundant supply of archival material for tumor diagnosis and staging via light microscopic evaluation. However, the current practice of specimen preparation is diverse and lacks strict standardization (thickness of tissue, volume of fixative, time of fixation) or well-defined standard operating procedures [37]. Accordingly, incomplete tissue fixation or tissue overfixation introduces significant sources of variability in the yield and quality of the nucleic acids that are extracted, resulting in suboptimal molecular analysis [38]. Although frozen tissue is the gold standard for molecular analyses, its use in pathological laboratories is impractical because of the associated expense and technical difficulty.

## 3. Experimental Section

### 3.1. Tissue Samples and Processing

CRC tissue samples (*n* = 131) were obtained surgically between 2006 and 2009 and were handled by the Department of Pathology (Montpellier, France). The institutional review board approved all of the protocols. To assess the feasibility of detecting *KRAS* mutations in both freshly frozen and FFPE tissues, the tissue samples were cut into two equal parts. One of the halves was immediately snap-frozen in liquid nitrogen and stored at −80 °C until DNA extraction. The other half was processed for formalin fixation and paraffin embedding using a TissueTek VIP automated processor (Bayer HealthCare Diagnosis Division). From the FFPE and frozen tissues, 7-μm-thick sections were cut and pooled into a 1.5-mL tube. A pathologist estimated the percentage of tumor cells for both the FFPE and frozen tissue sections.

### 3.2. DNA Isolation

All of the DNA was extracted using the DNA QIAamp DNA Extraction Kit (Qiagen) following the manufacturer’s instructions. For the FFPE tissues, the sections were dewaxed, followed by extraction in 100% xylene and washing with 100% ethanol. The samples are air-dried before DNA extraction. The extracted DNA was quantified using a NanoDrop ND-1000 Spectrophotometer (Nanodrop Technologies). The DNA integrity was assessed using 2% agarose gel electrophoresis.

### 3.3. PCR Amplification and DNA Sequencing

Two specific sets of oligonucleotide primers were used to determine the status of the mutations in exon 2 of *KRAS* in both the FFPE and frozen tissue sections. Set 1 (long fragment, 245 bp) included the forward and reverse primers 5′-GTACTGGTGGAGTATTTGAT-3′ and 5′-GTCCTGCACCAGTAATATGC-3′, respectively. Set 2 (short fragment, 164 bp) corresponded to the forward and reverse primers 5′-AAGGCCTGCTGAAAATGACTG-3′ and 5′-GTCCTGCACCAGTAATATGC-3′, respectively. Amplification was performed in a volume of 50 μL containing 1× PCR buffer, 250 μM deoxyribonucleoside triphosphate (dNTP), 0.4 μM of each forward and reverse primer, 5 units of AmpliTaq Gold^®^ DNA Polymerase (Applied Biosystems, Courtaboeuf, France), and 200 ng of genomic DNA. The thermal cycling conditions included a 10-min denaturation step at 94 °C, 40 cycles of 94 °C for 30 s, 60 °C for 30 s and 72 °C for 1 min, and a final extension at 72 °C for 7 min. The PCR products were run on an agarose gel and purified by exonuclease I digestion (Amersham Biosciences) and shrimp alkaline phosphatase (Roche Applied Sciences), according to the manufacturer’s instructions. Direct sequencing of the amplicons was performed with both the forward and the reverse primers using the BigDye™ Terminator v3.1 Cycle Sequencing Kit with the ABI PRISM™ 3100 Genetic Analyzer (Applied Biosystems). The mutations were confirmed by sequencing independent PCR products of DNA derived from tumor cells. All of the samples were analyzed in duplicate.

### 3.4. HRM Analysis

For the HRM screening, an 84-bp fragment from exon 2 of *KRAS* was PCR amplified using a Rotor-Gene 6000™ instrument (Qiagen) and the LightCycler 480 High Resolution Melting Master Reaction Mix (Roche Diagnostics). Each 20-μL reaction volume comprised of 25 ng purified genomic DNA, 10 μL reaction mix, 3.0 mmol/L MgCl_2_ and 0.25 μmol/L of each forward (5′GGCCTGCTGAAAATGACTGAA3′) and reverse (5′AATTAGCTGTATCGTCAAGGCACTC3′) primer. The cycling conditions were as follows: 95 °C for 5 min, followed by 50 cycles of 95 °C for 15 s, 63 °C for 25 s with an initial 11 cycles of touchdown (0.5 °C/cycle), and 72 °C for 25 s. The melting conditions included one cycle of 95 °C for 1 min, one cycle of 40 °C for 1 min and one cycle of 65 °C for 2 s, followed by a melt from 65 °C to 95 °C that increased 0.1 °C per second. All of the samples were tested in duplicate. The HRM data were analyzed using Rotor-Gene 6000 software (v1.7). For each sample, the normalized melting curves were evaluated, and these samples were compared with the wild-type sample controls in a deduced difference plot. Significant deviations from the horizontal line relative to the spread of the wild-type controls were indicative of sequence changes within the analyzed amplicon. The samples with distinct melting curves compared with the wild-type allele were recorded as positive mutations.

## 4. Conclusions

*KRAS* mutation is currently used to guide the clinical management of mCRC. Extreme caution must be taken when genotyping small amounts of DNA, especially if the DNA samples have been extracted from paraffin. In this study, we showed that the magnitude of agreement for the mutational status of *KRAS* between frozen and matched FFPE specimens was low, with suboptimal template amplifications and an erroneous wild-type genotype, regardless of the molecular method used. In addition, artifactual mutations in codons 12 and 13 of the *KRAS* gene from independent PCR amplification products were found to be associated with formalin specimen preservation. Finally, frozen specimen archiving is preferential where possible. When only FFPE samples are available, the risk of artifacts should be prevented by using large amounts of template DNA or by performing multiple amplifications. Alternatively, specimens may be fixed with both non-formalin and formalin fixatives.

## Figures and Tables

**Table 1.** Mutations in exon 2 of *KRAS* detected by HRM and sequencing in fresh-frozen samples.

**Nucleotide Change**	**Amino Acid Change**	**Number of Cases**
c.34G>A	p.G12S	2
c.34G>C	p.G12R	1
c.34G>T	p.G12C	1
c.35_36GT>TC	p.G12V	1
c.35G>A	p.G12D	15
c.35G>C	p.G12A	3
c.35G>T	p.G12V	13
c.37G>C	p.G13R	1
c.37G>T	p.G13C	1
c.38G>A	p.G13D	8
c.40G>A	p.V14I	1

**Total**		47

**Table 2.** Genotyping of exon 2 of *KRAS* in paired frozen and FFPE samples using HRM and direct sequencing.

**Sample no.**	**Frozen**			**FFPE**			
	
	Direct sequencing		HRM	Direct sequencing			HRM
	
	Nucleotide change	Amino acid change		Visual PCR band	Nucleotide change	Amino acid change	
	
8	c.35G>T	G12V	mutation	+	c.37G>T	G13C	mutation
11	c.35G>T	G12V	mutation	+	c.35G>T	G12V	mutation
13	c.35G>A	G12D	mutation	+	c.35G>A	G12D	mutation
18	c.34G>C	G12A	mutation	-	-	NA	mutation
21	c.35G>A	G12D	mutation	-	-	NA	mutation
24	c.35G>T	G12V	mutation	+	c.35G>T	G12V	mutation
25	c.35G>T	G12V	mutation	+	-	WT	NA
28	c.38G>A	G13D	mutation	+	-	WT	WT
29	c.35G>A	G12D	mutation	+	c.35G>T	G12V	mutation
32	c.35G>A	G12D	mutation	+	c.35G>A	G12D	mutation
36	c.35G>A	G12D	mutation	+	-	WT	mutation
38	c.34G>T	G12C	mutation	+	-	WT	mutation
42	c.35G>T	G12V	mutation	+	c.35G>T	G12V	mutation
43	c.35G>A	G12D	mutation	+	c.35G>A	G12D	mutation
65	c.34G>A	G12S	mutation	+	c.34G>A	G12S	mutation
68	c.38G>A	G13D	mutation	+	c.38G>A	G13D	mutation
74	c.38G>A	G13D	mutation	+	-	WT	mutation
79	c.35G>A	G12D	mutation	+	c.35G>A	G12D	mutation
80	c.35G>A	G12D	mutation	+	c.35G>A	G12D	mutation
84	c.35G>T	G12V	mutation	+	c.35G>T	G12V	mutation
91	c.35G>T	G12V	mutation	+	c.35G>T	G12V	mutation
96	c.37G>T	G13C	mutation	+	-	WT	NA
98	c.38G>A	G13D	mutation	+	c.38G>A	G13D	mutation
106	c.38G>A	G13D	mutation	+	c.38G>A	G13D	mutation
107	c.35G>A	G12D	mutation	+	c.35G>A	G12D	mutation
110	c.35G>A	G12D	mutation	+	c.35G>A	G12D	mutation
115	c.35G>T	G12V	mutation	+	c.35G>T	G12V	mutation
118	c.35G>T	G12V	mutation	+	c.35G>T	G12V	mutation
122	c.34G>C	G12A	mutation	+	c.34G>C	G12A	mutation
125	c.35_36GT>TC	G12V	mutation	+	c.35_36GT>TC	G12V	WT
127	c.35G>T	G12V	mutation	+	c.35G>A	G12D	mutation
129	c.35G>A	G12D	mutation	+	c.35G>A	G12D	mutation
131	c.35G>A	G12D	mutation	+	c.35G>A	G12D	mutation

WT, wild-type; NA, not amplified; 164 bp PCR product of a primer produced a visual band in the electrophoresis gel;

- , no visual band was detected.
